# Mitochondrial DNA Alterations in Glioblastoma (GBM)

**DOI:** 10.3390/ijms22115855

**Published:** 2021-05-29

**Authors:** Mariceli Baia Leão Barros, Danilo do Rosário Pinheiro, Bárbara do Nascimento Borges

**Affiliations:** 1 Molecular Biology Laboratory, Biological Sciences Institute, Federal University of Para, Belém, PA 66075, Brazil; maricelibaia@gmail.com; 2 Paragominas Campus, Federal Rural University of Amazonia, Paragominas, PA 68627, Brazil; danilopinheiro@ufra.edu.br

**Keywords:** mitochondrial DNA, glioblastoma, mtDNA alterations

## Abstract

Glioblastoma (GBM) is an extremely aggressive tumor originating from neural stem cells of the central nervous system, which has high histopathological and genomic diversity. Mitochondria are cellular organelles associated with the regulation of cellular metabolism, redox signaling, energy generation, regulation of cell proliferation, and apoptosis. Accumulation of mutations in mitochondrial DNA (mtDNA) leads to mitochondrial dysfunction that plays an important role in GBM pathogenesis, favoring abnormal energy and reactive oxygen species production and resistance to apoptosis and to chemotherapeutic agents. The present review summarizes the known mitochondrial DNA alterations related to GBM, their cellular and metabolic consequences, and their association with diagnosis, prognosis, and treatment.

## 1. Introduction

Grade IV glioma or glioblastoma (GBM) is the most common and aggressive malignant central nervous system (CNS) cancer in adults [1], accounting for 55% of all gliomas. It is associated with high rates of morbidity, relapse, and mortality [2,3,4] and can arise via a *de novo* pathway without clinical or histologic evidence of a less malignant precursor lesion (primary GBM), or via a progressive pathway through development from a low-grade astrocytoma (secondary GBM) [5,6].

Although age-related prognosis has no significant difference between the two histological types, primary GBM is frequently diagnosed at around 60 years of age and a more advanced stage, while secondary GBM is diagnosed at an early age, around 45 years [6]. One of the challenging features of GBM is its chemoresistance due to high biological complexity observed during tumorigenesis and progression [3,7].

Several GBM changes in mitochondrial function are observed at different levels, such as structural and functional changes, affecting mitogenic, hemodynamic, bioenergetic, and apoptotic signaling [8,9]. Altogether, these changes may indicate a decreased function of the oxidative phosphorylation system (OXPHOS) and energy linkage in glioma cells [10]. Thus, understanding the role of mitochondrial DNA (mtDNA) alterations in the development of glioblastoma carcinogenesis is extremely important [11]; hence, we provide a review of such phenomena.

## 2. Glioblastoma (GBM)

Gliomas are the most common primary brain tumors in adults [1,6,7,12], representing 28% of all brain neoplasms [1]. In 2020, 308,102 new cases of malignant brain tumors were diagnosed worldwide, especially in developed countries [13]. These tumors are characterized by their low relative incidence, poor prognosis, and the impairing of cognitive functions, consequently downgrading the life quality of patients [10].

Considering its histological origin, a glioma can be classified as a ependymoma, oligodendroglioma, astrocytoma, glioblastoma, or mixed glioma, among others [1]. According to the increased rate of de-differentiation and malignant nature, the World Health Organization (WHO) classified gliomas by different levels of malignancy from I to IV [6].

Astrocytomas, which develop from a subpopulation of cells with astrocytic differentiation, are the most common CNS tumors, classified as diffuse (WHO grade II), anaplastic (WHO grade III), or glioblastomas (WHO grade IV), of which the latter are further characterized as densely cellular, pleomorphic tumors with mitotic activity and either microvascular proliferation or necrosis, or both [6,14]. In 2016, the WHO reclassified tumors of the CNS, inserting glioblastomas in the group of diffuse astrocytic and oligodendroglial tumors due to their high degree of malignancy [15]. Besides, this new classification divided GBM into three classes: GBM IDH wild-type (about 90% of all cases), GBM IDH-mutant (about 10% of all cases), and GBM NOS (not otherwise specified) when IDH evaluation cannot be performed [15]. In humans, wild-types isocitrate dehydrogenase proteins IDH1 and IDH2, encoded by IDH1 (2q33) and IDH2 (15q26) genes, respectively [16], catalyze the oxidative decarboxylation of isocitrate in α-ketoglutarate (α-KG) and CO_2_ using NADP^+^ as a cofactor, resulting in NADPH formation. However, mutant isoforms IDH1 (R132H) and IDH2 (R172K) catalyze the conversion of α-KG to the oncometabolite D2-hydroxyglutarate (D2HG) [16,17,18,19,20] which inhibits α-KG-dependent dioxygenases, including the Jmjc-domain family of histone demethylases and the TET family of DNA dioxygenases, resulting in alterations of histone and DNA methylation, leading to a cellular environment that results in a malignant transformation [21,22,23].

Although with similar histology, GBM has several molecular differences; thus, a gene expression-based classification divides the tumor into four transcriptional subtypes: classical, neural, proneural, and mesenchymal, each one characterized by specific genetic and epigenetic modifications [6,24,25,26].

Later, several studies have considered only three transcriptional subtypes of GBM: proneural (PN), mesenchymal (MES), and classical (CL), which are associated not only with genetic and epigenetic signatures, but also with different prognostic and therapeutic implications and recurrence patterns [27,28,29,30]. Recently, it has been reported that a single GBM can present more than one transcriptional profile, contributing to the increasing transcriptional heterogeneity of the tumor [27,31].

Based on the combination of transcriptional data and immunohistochemistry (IHC), a new GBM classification was proposed, and taking into account the highly heterogeneous phenotype, the PN subtype was divided into two distinct forms: PN1 and PN2, the latter sharing several similarities with the CL subtype [29].

In general, the prognosis of glioblastoma is poor, with survival commonly less than two years [6,14]. Even considering the molecular classification of GBM, finding appropriate therapies remains a challenge, with few benefits on increased patient survival [32].

Currently, the main forms of treatment for GBM consist of surgery, chemotherapy with temozolomide, and radiotherapy. However, gliomas are within the category of cancers with difficult treatment, as they are resistant to conventional chemotherapy, diagnosed in advanced stages, and most have infiltrative growth, resulting in an incomplete surgical resection [3,7,26]. The unsatisfactory results of current therapies for gliomas are associated with the alteration of complex signaling pathways, the presence of the blood-brain barrier, and the existence of glioblastoma stem-like cells [33].

One of the reasons for the formation of therapy-resistant cells is the metabolic reprogramming, such as enhanced aerobic glycolysis (Warburg effect), as it results in cells that acquire a common set of properties, including high proliferation and resistance to apoptosis [3,28,29]. One of the main reasons that leads to the establishment of the Warburg effect, characterized by increased tumor cell glycolysis and decreased mitochondrial energy metabolism, even in the presence of oxygen, is tumoral hypoxia, a condition capable of maintaining and regulating stemness features through the activation of the NOTCH pathway, mediated by hypoxia-inducible factor-1α (HIF-1α) and 2α (HIF-2α) [34]. Besides, HIF-1α, as well as vascular endothelial growth factor (VEGF), may bind in hypoxia-responsible elements (HRE) located in the promoter region of the glycolytic enzyme hexokinase 2 (HK2), resulting in its up-regulated aberrant expression instead of its wild-type isoform hexokinase 1 (HK1), which helps the maintenance of the Warburg effect [35].

In this sense, it is known that mitochondria play a role in reprogramming the cellular metabolism [36], with metabolic flexibility serving to balance tumor cell energy needs with requirements for metabolites and precursors [37,38].

## 3. Mitochondria: Genome and Activity

Mitochondria are essential cellular organelles delimited by a double membrane system, external and internal (mitochondrial ridges), involved in numerous complex physiological processes, including cellular metabolism regulation, redox signaling, energy generation, regulation of cellular proliferation, and apoptosis [39]. As general characteristics, mitochondria have their own genetic material, and their biogenesis does not depend on the cell cycle [40].

In humans, mitochondrial DNA (mtDNA) has a circular double-stranded organization with multiple copies and is 16,569 bp in size [32,34,35]. The mitochondrial genome contains 13 protein-coding genes, responsible for the codification of the mitochondrial respiratory chain (MRC) and OXPHOS subunits (*ND1*, *ND2*, *ND3*, *ND4*, *ND4L*, *ND5*, *ND6*, *Cyt b*, *COI*, *COII*, *COIII*, *ATPase-6,* and *ATPase-8*), two ribosomal RNAs (rRNAs), and 22 transporter RNAs (tRNAs) genes [39]. mtDNA also has two non-coding regions called LOOP displacement (D-LOOP) and *OL*. The D-LOOP is the main mtDNA non-coding region where the origin of replication of the heavy (OH) strand and the sites promoting transcription for the light (LPS) and heavy (HSP1/2) strands of mtDNA are located [41]. On the other hand, the OL region is the source of light strand replication [32,40,42,43,44].

Mitochondria are, besides other processes, responsible for cell energy generation due to the presence of a series of multi-protein complexes (I–IV, forming the MRC) within the mitochondrial inner membrane, which generates reflux of protons used by complex V (ATP synthase) for the OXPHOS process to produce ATP and inorganic phosphate [14,45].

Electron transfers and proton translocations from the MRC and ATP generation from OXPHOS are related to several cellular processes, such as tricarboxylic acid cycle flow, regulation of nucleotide pools, generation of NADPH, carbon metabolism, ROS signaling, exchange ATP/ADP, calcium transport, protein import, apoptosis, inorganic phosphate transport, and mitochondrial membrane potential. Thus, this system has an important role in cell regulation as well as in molecular alterations in mitochondrial and/or nuclear genes, and thus, it could be related to the development and/or maintenance of diseases [10,38,39].

Most solid tumors, including glioma cells, behave according to the Warburg effect [14]. Therefore, tumor cells depend mainly on cytosolic ATP produced from glycolysis, rather than ATP derived from mitochondria [3,46].

## 4. Mitochondrial DNA Alterations in Glioblastoma

### 4.1. Mutations and Polymorphisms

Considering the substantial evidence supporting an involvement of mitochondria in the process of tumorigenesis, some studies have focused on the role of mtDNA changes in cancer development and maintenance. However, the precise underlying mechanism remains unknown [32,41,42].

Mutations in mtDNA can be found in about 60% of solid tumors, according to the International Cancer Genome Consortium (ICGC) and The Cancer Genome Atlas Program (TCGA), and could result in the coexistence of mixed mtDNA molecules in a single cell, a phenomenon known as heteroplasmy. These mutations occur in MRC subunits, the F_o_F_1_-ATPase subunit, or the protein biosynthesis on mitochondrial ribosomes, affecting ATP production and increasing oxidative stress [39]. Hence, understanding the mtDNA role in GBM development, progression, and drug resistance [32] is highly desirable because deregulation of mitochondrial function is considered a GBM marker [47].

Most mtDNA mutations are missense transitions, unequally distributed between HSP and LPS strands [48], as they are most likely to be functionally involved as modifiers of GBM tumorigenic processes [3]. Most somatic point mutations of mtDNA are present in the D-LOOP region, leading to dysregulations in replication and transcription processes, resulting in mtDNA copy number variations, mitochondrial dysfunction, and excessive production of ROS [3,47,49]; mutations associated with GBM can be found in almost all mtDNA genome (Table 1).

The D-LOOP region is the main hotspot for somatic mtDNA mutations, especially in a repeated sequence of cytosines between nucleotides 303 to 315, called polycytosine tract (poly-C), or D310 [41,50,51,53], which can be used for clinical follow-up of GBM [56]. Another D-LOOP hotspot polymorphism is T16189C, which although frequently observed, does not correlate with GBM etiology [49,50,51,54]. However, several less frequent alterations, like C16069T, T16126C, C16186T, G16274A, C16355T, and T16362C, were described as associated with GBM tumorigenesis [54].

Besides D-LOOP, codifying regions of mtDNA, especially those involved in MRC and OXPHOS, are also subject to mutations. Protein-coding genes of the MRC Complex I were the most affected, especially NADH dehydrogenase 4 (*ND4*) and NADH dehydrogenase 6 (*ND6*) genes. This suggests that such variants of the mitochondrial genome could offer advantage to the cells for promoting the tumorigenesis of glioblastoma [55]. One should notice that Complex I is involved in the cellular capacity to respond to oxygen deficiency and the establishment of hypoxia mechanisms; thus, genetic changes leading to its dysfunction can cause resistance to chemotherapeutic agents which need the redox cycle to be activated. For example, the T14634C mutation in *ND6* causes an amino acid change, resulting in a modification of the structure and orientation of the transmembrane helix of the ND6 protein [50].

The A10398G polymorphism in the NADH dehydrogenase 3 (*ND3*) gene results in a decrease of complex I function and, consequently, an increase of ROS cell production and oxidative stress, resulting in mtDNA damage, which can favor the onset and promotion of carcinogenesis [49].

Apart from being considered a hotspot for substitution mutations, genes involved in Complex I are also hotspots for other mutation types. As an example, it was observed that around 65% of the truncating somatic missense mutations of the mtDNA in GBM occurred in the NADH dehydrogenase 5 (*NAD5*) gene [57].

Alterations on Complexes III and IV coding genes are also frequent in GBM, some resulting in protein structural and functional alterations, considered as independent predictors of poor prognosis [58,61].

Recently, it was described that the T14798C mutation, which results in an amino acid change (F18L) in the central subunit of Cytochrome b (*Cyt b*), part of the MRC Complex III, can alter the activity and sensitivity to complex III-targeting drugs, altering ROS production, cell behavior, and the patient’s response to treatment, resulting in a poor prognosis. Thus, as a germline alteration, the T14798C mutation could be used as a non-invasive biomarker for prognostic and treatment response prediction [32]. Other mutations on *Cyt b* are related to Complex III activity or drug sensitivity. For example, G15257A and T14798C mutations increase drug sensitivity to atovaquone and clomipramine, respectively [60].

Even though most tumor- and non-tumor specific mtDNA mutations are not considered as deleterious events correlated with pathogenic processes, a small but important number of mutations on protein-coding genes may lead to alterations on mitochondrial function and might explain the metabolic plasticity observed on GBM [3].

### 4.2. Variation on MtDNA Content

Unlike the nuclear genome, mtDNA has several copies per cell as mitochondria undergo different dynamic processes, such as fusion and fission [62]. Even so, mtDNA content in each cell type is kept within a constant range to maintain cellular energy levels and, thus, cell function [59]. However, changes in mtDNA content are reportedly occurring at early stages of carcinogenesis [62,63], as a reflection of essential mutations for neoplastic transformation [62], leading to alterations in OXPHOS and, consequently, ATP production [64,65].

Currently, there are few studies about the influence of mtDNA copy number variation in GBM tumorigenesis, some with contradictory results. While mtDNA content maintenance during the differentiation process has been suggested [59,66], a higher mtDNA content was observed in patients with gliomas (GBM included) when compared to the control samples. Besides, a negative correlation between the increased mtDNA copy number and the histopathological grade of the glioma and tumor recurrence was also observed [47].

On the other hand, several studies have suggested a relationship between mtDNA content, GBM prognosis, and treatment response. However, in GBM cell lines (U87 and LN229), a low mtDNA copy number was associated with resistance to radiation and Temozolomide (TMZ) therapies [67] and with changes in nuclear genes methylation and expression patterns, leading to the expression of stemness markers [44,66]; studies using case-control samples observed a correlation between a high mtDNA copy number and the increase in overall survival [41,59,62], suggesting a better prognosis.

## 5. MtDNA Alterations and Liquid Biopsies

Currently, the diagnosis and monitoring of GBM patients consist, basically, of the use of neuroimaging approaches, such as magnetic resonance imaging (MRI) and computed tomography (CT) [68,69]. However, some limitations are related to those approaches, as imaging exams may not differentiate a true tumor progression from a pseudoprogression, leading to a misinterpretation of the patient’s response to therapy and delaying possible clinical interventions; additionally, tissue biopsies are extremely invasive and can cause cerebral edema and neurological disorders, which brings considerable risks to the patient [69,70]. Thus, the use of a more precise, less invasive strategies for the diagnosis, prognosis, molecular classification, response to treatment prediction, and assessment of GBM progression [68,71] are desirable. In recent years, the practice of liquid biopsy, fluid biopsy, or fluid phase biopsy have been proposed to overcome the limitations in the diagnosis and monitoring of GBM patients [70].

Liquid biopsies are based on the fact that tumors can eliminate several compounds in the blood circulation, such as circulating tumor cells (CTCs), cell-free nucleic acids (cfDNA and cfRNA), extracellular vesicles (EVs) [68,69,70,71,72], proteins [68,73], and tumor-educated metabolites and platelets (TEPs) [68], which can cross the blood-brain barrier [68,70] due to changes in its permeability [70,71].

Regarding cfDNA, which can be found freely, bound to proteins, or inside extracellular vesicles, it can be divided into two main classes: cell-free nuclear (cfnDNA) and mitochondrial (cfmtDNA) DNA, with the latter composed by generally short fragments, with 30 to 80 base pairs [74].

Body fluids such as blood, urine, saliva, and CSF can be used to detect cfmtDNA that are linked to GBM prognosis, response to treatment, and tumor recurrence [63,65,67]. In that sense, as tumoral mtDNA content variation is correlated with the patient’s prognosis, variations in mtDNA copy number in peripheral blood may be used as a biomarker for diagnosis [12,59] as well as prognosis [75].

## 6. Conclusions and Perspectives

Molecular alterations in mtDNA can deregulate mitochondria function. In that sense, despite several mtDNA mutations that have been described in GBM, only a few, located in protein-coding genes related with OXPHOS, have been considered deleterious, resulting in alteration of the energy-production process and in abnormal cell function, which can lead to resistance to some chemotherapeutic agents. However, due to a lack of characterization of their functional consequences, their real role in GBM tumorigenesis has not yet been determined. Considering this, the utilization of mtDNA mutations and polymorphisms in clinical routine as GBM prognosis biomarkers, although promising, should be carefully considered.

On the other hand, alterations on mtDNA content, as a result or not from mutations on the D-LOOP region, support their use as a diagnostic, prognostic, and response-to-treatment marker, making them a promising biomarker mainly for non-invasive approaches, such as liquid biopsy. In this respect, the mtDNA content quantification may be useful, especially in monitoring GBM recurrences in a less invasive and less stressful way for the patients.

## Figures and Tables

**Table 1:** Mitochondrial DNA alterations in GBM.

Region/Gene	Variation	Type	Amino Acid (aa) Change	Reference
*D-LOOP*	Np61 C→A	Transversion	-	[49]
	Np64 C→T	Transition	-	[50]
	Np71	G-deletion	-	[49]
	Np72 T→C	Transition	-	[51]
	Np73 A→G	Transition	-	[44,47,48]
	Np146 T→C	Transition	-	[49,52]
	Np150 C→T	Transition	-	[53]
	Np152 T→C	Transition	-	[49,50]
	Np152 A→G	Transition	-	[54]
	Np176	A-deletion	-	[49]
	Np185 G→A	Transition	-	[53]
	Np185 A→G	Transition	-	[51]
	Np186 C→G	Transition	-	[49]
	Np189 A→G	Transition	-	[53]
	Np194 T→C	Transition	-	[55]
	Np195 T→C	Transition	-	[49,50,51,52,54]
	Np204 T→C	Transition	-	[53]
	Np 204 C→T	Transition	-	[44,46,47,49]
	Np207 G→A	Transition	-	[54]
	Np249	A-deletion	-	[49]
	Np263 A→G	Transition	-	[44,45,47,49]
	Np295 C→T	Transition	-	[51]
	Np302 A→C	Transversion	-	[55]
	Np303	CC-insertion	-	[49]
	Np303–309	C-insertion	-	[50]
	Np310 T→C	Transition	-	[55]
	Np310 (C_8_TC_6_)→(C_9_TC_6_)	Insertion	-	[51]
	Np310 (C_7_TC_6_)→(C_9_TC_6_)	Insertion	-	[51]
	Np311	CC-insertion	-	[49]
	Np311–315	C-insertion	-	[50]
	Np320 C→T	Transition	-	[50]
	Np343 C→G	Transversion	-	[50]
	Np411 C→G/C	Heteroplasmy	-	[49]
	Np476 C→A	Transversion	-	[49]
	Np489 T→C	Transition	-	[54]
	Np514 (CA)_4_→(CA)_6_	Insertion	-	[51]
	Np523–524	AC-deletion	-	[49]
	Np566 C→A	Transversion	-	[49]
	Np16069 C→T	Transition	-	[54]
	Np16126 T→C	Transition	-	[51,54]
	Np16129 G→A	Transition	-	[50]
	Np16134 C→T	Transition	-	[51]
	Np16145 G→A	Transition	-	[54]
	Np16148 C→T	Transition	-	[54]
	Np16172 T→C	Transition	-	[54]
	Np16186 C→T	Transition	-	[54,55]
	Np16189 T→C	Transition	-	[49,50,51,54]
	Np16189 (C)_10_→(C)_11_	Insertion	-	[51]
	Np16189 (C)_10_→(C)_9_	Deletion	-	[51,52]
	Np16189 (C)_12_→(C)_11_	Deletion	-	[51]
	Np16193 C→T	Transition	-	[54]
	Np16218 C→T	Transition	-	[55]
	Np16220 A→C	Tranversion	-	[50]
	Np16223 C→T	Transition	-	[54]
	Np16224 T→C	Transition	-	[54,55]
	Np16234 C→T	Transition	-	[54]
	Np16256 C→T	Transition	-	[54]
	Np16261 C→T	Transition	-	[49,54]
	Np16264 C→T	Transition	-	[50]
	Np16265 A→G	Transition	-	[49]
	Np16270 C→T	Transition	-	[49,54]
	Np16274 G→A	Transition	-	[54]
	Np16292 C→T	Transition	-	[54]
	Np16293 A→G	Transition	-	[51]
	Np16311 T→C	Transition	-	[54,56]
	Np16316 A→G	Transition	-	[50]
	Np16318 A→C	Tranversion	-	[54]
	Np16327 C→T	Transition	-	[54]
	Np16336 T→C	Transition	-	[56]
	Np16355 C→T	Transition	-	[54]
	Np16356 T→C	Transition	-	[49,51]
	Np16362 T→C	Transition	-	[54]
	Np16381 T→C	Transition	-	[49]
	Np16384 G→A	Transition	-	[54]
	Np16392 G→A	Transition	-	[54]
	Np16394 G→A	Transition	-	[54]
	Np16405	A-deletion	-	[49]
	Np16438 G→A	Transition	-	[56]
	Np16477 G→A	Transition	-	[54]
	Np16519 T→C	Transition	-	[49,50,51,54,55]
	Np16524 A→G	Transition	-	[56]
	Np16526 G→A	Transition	-	[56]
	Np16527 C→T	Transition	-	[56]
16S rRNA	Np1913 G→A	Transition	-	[3]
	Np1966 G→A	Transition	-	[57]
	Np2002 G→A	Transition	-	[55]
	Np2130 A→G	Transition	-	[55]
	Np2652 G→A	Transition	-	[3]
	Np2817 G→A	Transition	-	[55]
	Np2976 G→A	Transition	-	[3]
	Np3036 G→A	Transition	-	[57]
	Np3168 C→T	Transition	-	[55]
tRNA^Gln^	Np4395 A→G	Transition	-	[3]
tRNA^Met^	Np4403 G→A	Transition	-	[3]
tRNA	Np596 T→C	Transition	-	[3]
tRNA	Np15928 G→A	Transition	-	[54]
tRNA	Np15936 T→C	Transition	-	[54]
12S rRNA	Np750 A→G	Transition	-	[50,54]
	Np1227 G→A	Transition	-	[57]
	Np1386 T→C	Transition	-	[55]
	Np1415 G→A	Transition	-	[57]
L strand replication origin	Np5752 A→G	Transition	-	[55]
*ATPase 6*	Np8528 T→C	Transition Nonsynnonymous	Met→Thr (M1T)	[3]
	Np8603 T→C	Transition Nonsynnonymous	Phe→Ser (F26S)	[3]
	Np8632 T→C	Transition Nonsynnonymous	Tyr→His (Y36H)	[3]
	Np8668 T→C	Transition Nonsynnonymous	Trp→Arg (W48R)	[3]
	Np8860 A→G	Transition Nonsynnonymous	Thr→Ala (T112A)	[50]
	Np8919 A→G	Transition Nonsynnonymous	Gln→Gln (Q131Q)	[50]
	Np9025 G→A	Transition Nonsynnonymous	Gly→Ser (G167S)	[3]
	Np9139 G→A	Transition Nonsynnonymous	Ala→Thr (A205T)	[3]
*ATPase 8*	Np8496 T→C	Transition Nonsynnonymous	Met→Thr (M44T)	[3]
	Np8462 T→C	Transition Nonsynnonymous	Tyr→His (Y33H)	[3]
*ND1*	Np3344 T→C	Transition Nonsynnonymous	Ile→Thr (I13T)	[57]
	Np3357 G→C	Transversion Nonsynnonymous	Met→Ile (M17I)	[3]
	Np3538 G→A	Transition Nonsynnonymous	Ala→Thr (A78T)	[3]
	Np3631 T→A	Transversion Nonsynnonymous	Ser→Thr (S109T)	[3]
	Np3866 T→C	Transition Nonsynnonymous	Ile→Thr (I187T)	[3]
	Np4029 C→A	Transversion Nonsynnoymous	Ile→Met (I241M)	[3]
	Np4136 A→G	Transition Nonsynnoymous	Tyr→Cys (Y277C)	[3]
*ND2*	Np4646 T→C	Transition Synnonymous	Tyr→Tyr (Y59Y)	[51]
	Np4752 T→C	Transition Nonsynnoymous	Ser→Pro (S95P)	[3]
	Np4762 T→C	Transition Nonsynnonymous	Ile→Thr (I98T)	[57]
	Np4769 A→G	Transition Synnonymous	Met→Met (M100M)	[50]
	Np5198 A→G	Transition Synnonymous	Leu→Leu (L243L)	[51]
	Np5263 C→T	Transition Nonsynnoymous	Ala→Val (A265V)	[3]
*ND3*	Np10398 A→G	Transition Nonsynnoymous	Thr→Ala (T114A)	[49]
*ND4L*	Np10586 G→A	Transition Synnonymous	Ser→Ser (S39S)	[57]
	Np10473 C→G	Tranversion Nonsynnoymous	Pro→Ala (P2A)	[55]
*ND4*	Np10814 A→C	Tranversion Nonsynnoymous	Lys→Gln (K19Q)	[55]
	Np10946	C-insertion	Frameshift	[3]
	Np11084 A→G	Transition Nonsynnoymous	Thr→Ala (T109A)	[3]
	Np11711 G→A	Transition Nonsynnoymous	Ala→Thr (A318T)	[3]
	Np11718 G→A	Transition Nonsynnoymous	Gly→Glu (G320E)	[3]
	Np11719 G→A	Transition Synnoymous	Gly→Gly (G320G)	[50]
	Np11361 T→C	Transition Nonsynnoymous	Met→Thr (M201T)	[55]
	Np11512 C→A	Tranversion Nonsynnoymous	Asn→Lys (N251K)	[55]
	Np11674 C→T	Transition Synnonymous	Thr→Thr (T305T)	[55]
	Np11693 G→A	Transition Nonsynnoymous	Ala→Thr (A312T)	[3]
	Np11866	C-insertion	Frameshift	[3]
	Np11928 A→G	Transversion Nonsynnoymous	Asn→Ser (N390S)	[3]
	Np11978 T→A	Transition Nonsynnoymous	Ser→Thr (S407T)	[3]
	Np12033 A→G	Transition Nonsynnoymous	Asn→Ser (N425S)	[3]
	Np12101 T→C	Transition Nonsynnoymous	Ser→Pro (S448P)	[55]
	Np12102 C→T	Transition Nonsynnoymous	Ser→Phe (S448F)	[55]
*ND5*	Np12418	A-deletion	Frameshift	[3]
	Np12936 A→G	Transition Synnonymous	Gln→Gln (Q200Q)	[51]
	Np12877 G→C	Transversion Nonsynnoymous	Gly→Arg (G181R)	[55]
	Np13061 C→A	Transversion Nonsynnoymous	Pro→Gln (P242Q)	[55]
	Np13043 C→T	Transition Nonsynnoymous	Ala→Val (A236V)	[55]
	Np13063 G→A	Transition Nonsynnoymous	Val→Ile (V243I)	[3]
	Np13112 T→C	Transition Nonsynnoymous	Leu→Ser (L259S)	[3]
	Np13124 T→C	Transition Nonsynnoymous	Phe→Ser (F263S)	[51]
	Np13135 G→A	Transition Nonsynnoymous	Ala→Thr (A267T)	[3]
	Np13468 C→A	Transvertion Nonsynnoymous	Leu→Met (L378M)	[3]
	Np13613 T→C	Transition Nonsynnoymous	Met→Thr (M426T)	[3]
	Np13835 C→T	Transition Nonsynnoymous	Thr→Met (T500M)	[3]
	Np13858 A→G	Transition Nonsynnoymous	Thr→Ala (T508A)	[3]
	Np13879 T→C	Transition Nonsynnoymous	Ser→Pro (S515P)	[3]
*ND6*	Np14159 C→G	Transversion Nonsynnoymous	Arg→Pro (R172P)	[55]
	Np14160 G→C	Transversion Nonsynnoymous	Arg→Gly (R172G)	[55]
	Np14180 T→C	Transition Nonsynnoymous	Tyr→Cys (Y165C)	[3]
	Np14426 C→T	Transition Nonsynnoymous	Gly→Glu (G85E)	[55]
	Np14470 T→C	Transition Synnonymous	Gly→Gly (G68G)	[50]
	Np14620 C→T	Transition Synnonymous	Gly→Gly (G18G)	[51]
	Np14634 T→C	Transition Nonsynnoymous	Met→Val (M14V)	[50]
*CYTB*	Np14766 C→T	Transition Nonsynnoymous	Thr→Ile (T7I)	[58]
	Np14793 A→G	Transition Nonsynnoymous	His→Arg (H16R)	[58]
	Np14798 C→T	Transition Nonsynnoymous	Phe→Leu (F18L)	[25,45,46,52,53]
	Np14814 C→T	Transition Nonsynnoymous	Thr→Ile (T23I)	[3]
	Np15042 G→A	Transition Nonsynnoymous	Gly→Glu (G99E)	[3]
	Np15071 T→C	Transition Nonsynnoymous	Tyr→His (Y109H)	[3]
	Np15168 G→A	Transition	Premature Stop Codon	[3]
	Np15048 G→C	Transition Nonsynnoymous	Gly→Ala (G101A)	[58,59]
	Np15218 A→G	Transition Nonsynnoymous	Thr→Ala (T158A)	[58]
	Np15257 G→A	Transition Nonsynnoymous	Asn→Asp (N171D)	[58,60]
	Np15264 C→T	Transition Nonsynnoymous	Pro→Leu (P173L)	[55]
	Np15267 C→G	Transversion Nonsynnoymous	Thr→Ser (T174S)	[55]
	Np15326 A→G	Transition Nonsynnoymous	Thr→Ala (T194A)	[50,58]
	Np15375 G→A	Transition Nonsynnoymous	Gly→Glu (G210E)	[3]
	Np15452 C→A	Transversion Nonsynnoymous	Leu→Ile (L236I)	[58]
	Np15453 T→C	Transition Nonsynnoymous	Leu→Pro (L236P)	[58]
	Np15479 T→C	Transition Nonsynnoymous	Phe→Leu (F245L)	[58]
	Np15500 G→A	Transition Nonsynnoymous	Asp→Asn (D252N)	[58,60]
	Np15506 G→A	Transition Nonsynnoymous	Asp→Asn (D254N)	[3]
	Np15693 T→C	Transition Nonsynnoymous	Met→Thr (M316T)	[58]
	Np15758 A→G	Transition Nonsynnoymous	Ile→Val (I338V)	[58]
	Np15773 G→A	Transition Nonsynnoymous	Val→Met (V343M)	[3]
	Np15803 G→A	Transition Nonsynnoymous	Val→Met (V353M)	[58]
	Np15884 G→C	Transversion Nonsynnoymous	Ala→Pro (A380P)	[54]
*COI*	Np5999 T→C	Transition Synnonymous	Ala→Ala (A32A)	[51]
	Np6047 A→G	Transition Synnonymous	Leu→Leu (L48L)	[51]
	Np6111 G→A	Transition Nonsynnoymous	Val→Met (V70M)	[3]
	Np6180 G→A	Transition Nonsynnoymous	Ala→Thr (A93T)	[3]
	Np6237 C→A	Transversion Nonsynnoymous	Leu→Met (L112M)	[58]
	Np6267 G→A	Transition Nonsynnoymous	Ala→Thr (A122T)	[58]
	Np6422 C→T	Transition Synnonymous	Pro→Pro (P173)	[55]
	Np6619 G→A	Transition Nonsynnonymous	Gly→Asp (G239D)	[57]
	Np6692	A-deletion	(M271Ter)	[58]
	Np6709 G→A	Transition Nonsynnoymous	Gly→Glu (G269E)	[3]
	Np6999 G→A	Transition Nonsynnoymous	Val→Met (V366M)	[55]
	Np7080 T→C	Transition Nonsynnoymous	Phe→Leu (F393L)	[3]
	Np7153 T→C	Transition Nonsynnoymous	Met→Thr (M417T)	[3]
*COII*	Np7919 G→A	Transition Nonsynnoymous	Asp→Asn (D112N)	[3]
	Np7965 T→C	Transition Nonsynnoymous	Phe→Ser (F127S)	[3]
	Np7976 G→A	Transition Nonsynnoymous	Gly→Ser (G131S)	[3]
	Np8251 G→A	Transition Synnonymous	Gly→Gly (G222G)	[55]
	Np8252 C→A	Tranversion Nonsynnoymous	Pro→Thr (P223T)	[55]
*COIII*	Np9252 T→C	Transition Nonsynnoymous	Trp→Arg (W16R)	[58]
	Np9276 G→A	Transition Nonsynnoymous	Ala→Thr (A24T)	[3]
	Np9280 T→C	Transition Nonsynnoymous	Leu→Pro (L25P)	[3]
	Np9300 G→A	Transition Nonsynnoymous	Ala→Thr (A32T)	[58]
	Np9438 G→A	Transition Nonsynnoymous	Gly→Ser (G78S)	[3]
	Np9469 C→T	Transition Nonsynnoymous	Thr→Ile (T88I)	[58]
	Np9477 G→A	Transition Nonsynnoymous	Val→Ile (V91I)	[58]
	Np9627 G→A	Transition	Premature stop codon	[3]
	Np9655 G→A	Transition Nonsynnonymous	Ser→Asn (S150N)	[57,58]
	Np9667 A→G	Transition Nonsynnonymous	Asn→Ser (N154S)	[58]
	Np9786 G→A	Transition Nonsynnonymous	Gly→Ser (G194S)	[3]
	Np9966 G→A	Transition Nonsynnoymous	Val→Ile (V254I)	[58]

## Data Availability

Not applicable.
